# Mechanical properties of NiTi and CuNiTi wires used in orthodontic treatment. Part 2: Microscopic surface appraisal and metallurgical characteristics

**DOI:** 10.1590/2176-9451.19.1.069-076.oar

**Published:** 2014

**Authors:** Marco Abdo Gravina, Cristiane Canavarro, Carlos Nelson Elias, Maria das Graças Afonso Miranda Chaves, Ione Helena Vieira Portella Brunharo, Cátia Cardoso Abdo Quintão

**Affiliations:** 1 Adjunct professor, Department of Orthodontics, UFJF.; 2 Assistant professor, Department of Orthodontics, FOUERJ.; 3 Assistant professor, Military Institute of Engineering (IME).; 4 Adjunct professor, Department of Pathology, UFJF.; 5 Assistant professor, Department of Orthodontics, FOUERJ.; 6 Adjunct professor, Department of Orthodontics, FOUERJ.

**Keywords:** Physical properties, Orthodontic wires, Scanning electron microscopy, Nickel, Titanium, Copper

## Abstract

**Objective:**

This research aimed at comparing the qualitative chemical compositions and the surface morphology of fracture regions of eight types of Nickel (Ni) Titanium (Ti) conventional wires, superelastic and heat-activated (GAC, TP, Ormco, Masel, Morelli and Unitek), to the wires with addition of copper (CuNiTi 27ºC and 35ºC, Ormco) after traction test.

**Methods:**

The analyses were performed in a scanning electronic microscope (JEOL, model JSM-5800 LV) with EDS system of microanalysis (energy dispersive spectroscopy).

**Results:**

The results showed that NiTi wires presented Ni and Ti as the main elements of the alloy with minimum differences in their composition. The CuNiTi wires, however, presented Ni and Ti with a significant percentage of copper (Cu). As for surface morphology, the wires that presented the lowest wire-surface roughness were the superelastic ones by Masel and Morelli, while those that presented the greatest wire-surface roughness were the CuNiTi 27ºC and 35ºC ones by Ormco, due to presence of microcavity formed as a result of pulling out some particles, possibly of NiTi.^4^ The fracture surfaces presented characteristics of ductile fracture, with presence of microcavities. The superelastic wires by GAC and the CuNiTi 27ºC and the heat-activated ones by Unitek presented the smallest microcavities and the lowest wire-surface roughness with regard to fracture, while the CuNiTi 35ºC wires presented inadequate wire-surface roughness in the fracture region.

**Conclusion:**

CuNiTi 35ºC wires did not present better morphologic characteristics in comparison to the other wires with regard to surfaces and fracture region.

## Abstract (pt)

**Objetivo:**

objetivou-se nessa pesquisa comparar as composições químicas qualitativas e as morfologias das superfícies e das regiões de fratura, após ensaios de tração, de oito tipos de fios de NiTi superelásticos e termoativados convencionais (GAC, TP, Ormco, Masel, Morelli e Unitek) aos fios com adição de cobre (CuNiTi 27°C e 35°C, da Ormco).

**Métodos:**

as análises foram realizadas em microscópio eletrônico de varredura (JEOL, modelo JSM-5800 LV) com sistema de microanálise EDS (*energy dispersive spectroscopy*).

**Resultados:**

os resultados mostraram que os fios de NiTi ensaiados apresentaram Ni e Ti como os principais elementos da liga, com diferenças mínimas em suas composições. Os fios de CuNiTi, por sua vez, apresentaram Ni e Ti, com percentual significativo de cobre (Cu). Em relação às morfologias das superfícies, os fios que apresentaram os melhores acabamentos foram os superelásticos da Masel e Morelli, e os que apresentaram os piores acabamentos foram os de CuNiTi 27°C e 35°C, da Ormco, graças à presença de microcavidades formadas devido ao arrancamento de partículas, possivelmente de NiTi^4^. As superfícies de fratura apresentaram características de fratura dúctil, com a presença de microcavidades. Os fios superelásticos da GAC e de CuNiTi 27°C, bem como os termoativados da Unitek, apresentaram as menores microcavidades e os melhores acabamentos à fratura, enquanto os fios de CuNiTi 35° apresentaram acabamentos inadequados das regiões de fratura.

**Conclusão:**

os fios de CuNiTi 35°C não apresentaram características morfológicas mais adequadas das superfícies e das regiões de fratura em relação aos demais fios.

## INTRODUCTION

Since NiTi wires were first supplied in the orthodontic market, over thirty years ago, improvements have been obtained in order to enhance its performance in dental leveling and alignment. In 1963, the NiTi alloys were developed at the Naval Ordinance Laboratory of Silver Springs, Maryland, USA. In orthodontic practice, Andreasen et al^1^ were attracted by the unique properties inherent to these alloys, such as high elastic limit, high resilience and low module of elasticity and rigidity. For this reason, they developed the first commercial NiTi alloys aimed at orthodontic purposes.^1^ Subsequently, Unitek Corporation produced the stabilized NiTi alloy for clinical use under the trade name Nitinol (Nickel Titanium Naval Ordinance Laboratory). It was developed on the weight percentage of 55% of nickel and 45% of titanium, and clinically used for the first time in 1972. Despite its excellent property of elastic recovery, Nitinol did not have, by that time, shape memory or superelasticity.

Superelastic NiTi alloys (SE NiTi) were initially produced in 1978 by Furukawa Eletric Co. (Japan). Thereafter, several studies were carried out in the attempt to produce wires with similar properties for orthodontic purposes, which was achieved in 1986, with the development of the Japanese NiTi wire. Concomitantly to this discovery, another NiTi alloy with similar characteristics was developed specially for orthodontic purposes by Tien Hua Cheng at the General Research Institute for Non-Ferrous Metals (Beijing, China).^5^ These alloys, named Chinese NiTi, had unique characteristics of constant maintenance of forces during activation and deactivation.^5,11^ The austenitic crystal structure obtained during the manufacturing process significantly differs them from stabilized NiTi wires.^5^

Heat-activated NiTi alloys emerged, for commercial purposes, in the 90's. In addition to the property of maintaining the constant load of superelastic wires, thermodynamic NiTi wires had the additional characteristic of being thermally activatable, a property that is responsible for shape memory.^11^ Since 1963, Buehler^3^ has observed the shape memory effect^14^, asserting that, in low temperatures, the NiTi alloys were easily deformed and, when heating them above their transition temperatures, their original configurations were re-established. However, the complexity of the thermic treatment of NiTi alloys made it impossible, at that time, to manufacture heat-activated wires.^7,16^ NiTi wires with copper (CuNiTi) emerged, commercially, in the mid 90's and were manufactured in three transition temperatures, one of which was superelastic (CuNiTi 27ºC) and two heat-activated (CuNiTi 35ºC and CuNiTi 40ºC). Due to having copper (efficient heat conductor) added to nickel and titanium, these wires present better defined transition temperatures, which not only ensures the generation of more homogeneous loadings from arch to arch and from end to end, but also increases the effectiveness of tooth movement.^15^

Fischer-Brandies et al^9^ compared the chemical composition and the superficial topography of five trade brands of rectangular superelastic NiTi wires (Dentaurum, Forestadent and Lancer) and heat-activated wires (GAC and CuNiTi 35ºC). The results showed that all tested wires presented minimum differences in their chemical composition, with nearly 58% of nickel and 42% of titanium, except for the CuNiTi wire which, besides nickel (50.7%) and titanium (42.4%), presented 6.9% of copper in its composition. With regard to superficial topography, all wires presented inclusion and manufacturing residue (particles of Si and Al), chemical immunogeneicities and superficial slots. Superelastic wires presented smoother surfaces in comparison to the heat-activated ones. Such characteristics are important because they are associated with the intensity of bracket-wire attrition, biocompatibility and resistance to corrosion, given that the release of ions and the fractures caused by fatigue start on the spot of inclusion and corrosion.^9^

Damon^6^ stated that the specific use of CuNiTi 35ºC orthodontic wires associated with self-bonding brackets (Damon system) would significantly reduce the coefficient of attrition generated in conventional mechanics, which is the key to an efficient treatment. As a result, there would be significant reduction in the mean period of chair time, the number of visits paid to the orthodontist and in patient's degree of discomfort.

In this context, the present study aimed at: (1) carrying out and comparing qualitative analyses of the chemical composition of superelastic and heat-activated NiTi and CuNiTi wires of different trade brands, through scanning electron microscopy (SEM) with EDS system of microanalysis (energy dispersive spectroscopy); and (2) analyzing and comparing by means of SEM and the technique of secondary electrons analysis, the surface finishing as well as the fracture region of these wires, given that such characteristics could affect the clinical performance of these alloys and, more specifically, the coefficient of attrition, the biocompatibility and the adequate resistance to corrosion and to fracture under orthodontic forces.^2,4,10,12^

## MATERIAL AND METHODS

Twenty orthodontic 0.018" gauge precontoured wires were used, all of which had the same arch shape and were commercialized by six different companies. The wires were divided into two groups (superelastic and heat-activated) according to their mechanical properties which were informed by the manufacturers. Groups and subgroups of the analyzed samples are shown in Table 1. The wires were subjected to traction tests of which results are presented in part 1 of this work.^17^

**Table 1:** Orthodontic wires used in this study: GAC (GAC Int. Inc. New York, USA); TP (TP Orthodontics, La Porte, USA); Ormco (Ormco Corp. Glendora, USA); 3M Unitek (Unitek Corporation, Monrovia, USA); Masel (Masel, Bristol, USA); Morelli (Dental Morelli, São Paulo, Brazil).

SUPERELASTIC GROUP (12 archwires)		Heat-ACTIVATED GROUP (08 archwires)	
GAC	2 NiTi SE (REF. 03-018-53T)	GAC Sentalloy	2 NiTi Heat (REF. 02-511-132)
TP Reflex	2 NiTi SE (REF. 381-264)	TP HA	2 NiTi Heat (REF. 381-825)
Ormco Ni-Ti	2 NiTi SE (REF. 219-3204)	Ormco 35OC	2 CuNiTi Heat (REF. 219-4204)
Ormco 27°C	2 CuNiTi SE (REF. 205-0048)	Unitek Nitinol HA	2 NiTi Heat (REF. 4286-981)
Masel Elastinol	2 NiTi SE (REF. 4828-018)	-	-
Morelli	2 NiTi SE (REF. 50.70.014)	-	-

**Figure 1:**
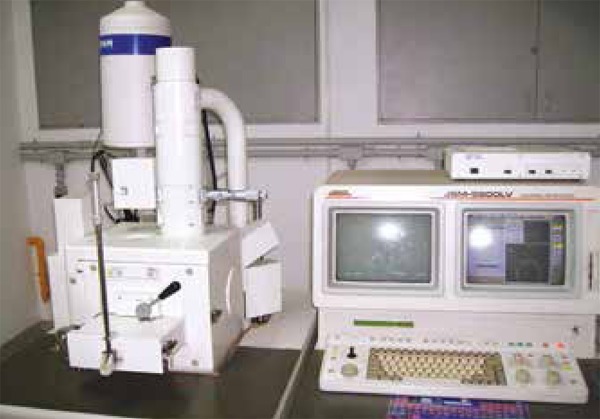
Scanning electronic microscope used with EDS system of microanalysis.

The semiquantitative chemical composition was determined by the same methodology used by Fischer-Brandies et al.^9^ Surface morphology and fracture regions of the wires were analyzed by scanning electron microscopy (JEOL, model JSM-5800 LV with EDS system of microanalysis) (Fig 1). To analyze the surface morphology of the fracture region and wire-surface roughness of the wires, the technique of secondary electrons analysis was used, by means of which the images of inclusions, manufacturing residue and superficial heterogeneity were obtained. To carry out these tests, two samples of each wire subgroup were used.

## RESULTS

### Semiquantitative chemical composition

Chemical analyses revealed that all samples basically have Ni and Ti with minor differences in percentage. CuNiTi wires presented significant percentage of copper in addition to Ni and Ti in their composition. Microanalyses results shown in Table 2 indicate the oxides present on the surface of the analyzed wires.

**Table 2:** Chemical composition of tested NiTi and CuNiTi wires (EDS).

Chemical composition %	Nickel (Ni) NiO	Titanium (Ti) TiO_2_	Copper (Cu) CuO	Aluminium (Al) Al_2_O_3_	Calcium (Ca) CaO	Bromine (Br) Br	Silicon (Si) SiO_2_
NiTi SUPER GAC	53.33%	43.52%	–	0.19%	–	–	–
NiTi SUPER TP	52.50%	45.44%	–	1.61%	0.16%	–	0.20%
CuNiTi 27oC Ormco	46.87%	43.70%	6.72%	2.31%	–	–	–
NiTi SUPER Ormco	50.99%	42.27%	–	–	3.50%	0.54%	1.98%
NiTi SUPER Masel	49.22%	39.40%	–	5.08%	0.27%	4.26%	1.42%
NiTi SUPER Morelli	53.84%	42.96%	–	2.11%	–	–	0.58%
NiTi Heat GAC	52.84%	41.61%	–	1.13%	0.27%	–	1.59%
NiTi Heat TP	53.48%	43.26%	–	0.86%	–	–	–
CuNiTi 35oC Ormco	43.82%	45.79%	5.09%	2.40%	0.99%	–	0.69%
NiTi Heat Unitek	54.08%	45.14%	–	0.32%	–	–	0.23%

## Wire-surface roughness

**Figure 2:**
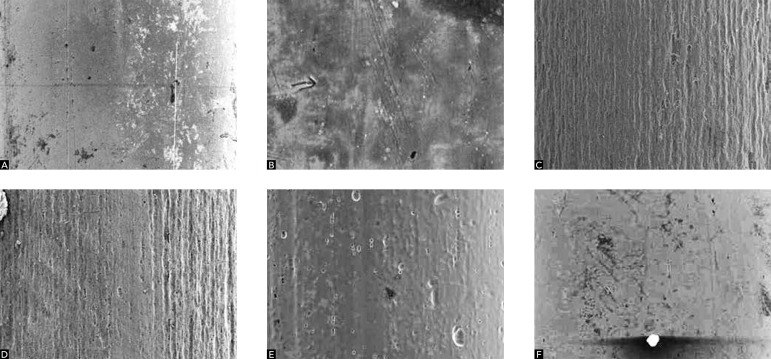
Surface morphologies of superelastic NiTi and CuNiTi wires: A) superelastic NiTi by GAC; B) superelastic NiTi by TP; C) superelastic NiTi by Ormco; D) CuNiTi 27oC by Ormco; E) superelastic NiTi by Masel; F) superelastic NiTi by Morelli (X 500).

**Figure 3:**
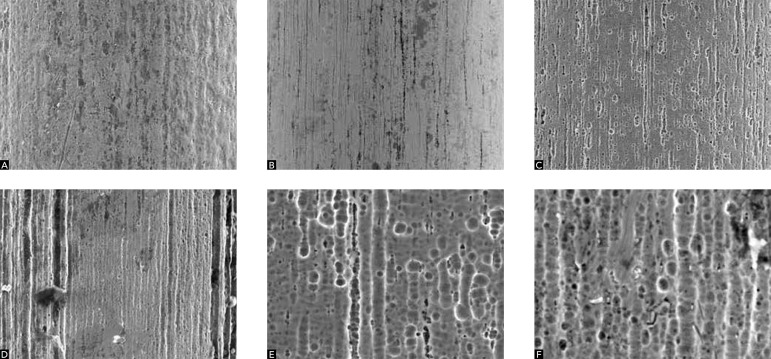
Surface morphologies of heat-activated NiTi and CuNiTi wires: A) heat-activated NiTi by GAC; B) heat-activated NiTi by TP; C) CuNiTi 35oC by Ormco; D) heat-activated NiTi by Unitek (X500); E) CuNiTi 35oC by Ormco; F) heat-activated NiTi by Unitek (X 2000).

Figures 2 and 3 show, respectively, the surface morphology of superelastic and heat-activated wires before traction tests. It can be observed that superelastic NiTi wires presented lower wire-surface roughness than the heat-activated ones.

## Surface morphology of wire fracture

**Figure 4:**
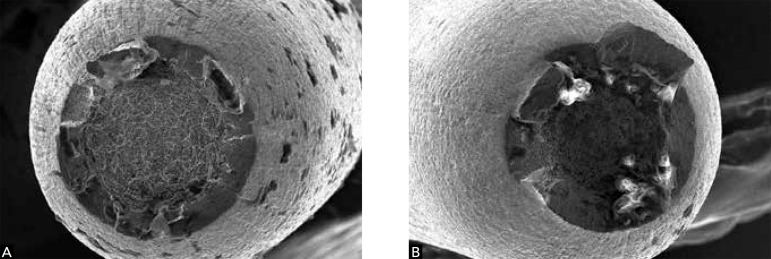
Scanning electron microscopy of the fracture region on A) CuNiTi 27oC by Ormco and B) heat-activated NiTi by GAC (X 250).

**Figure 5:**
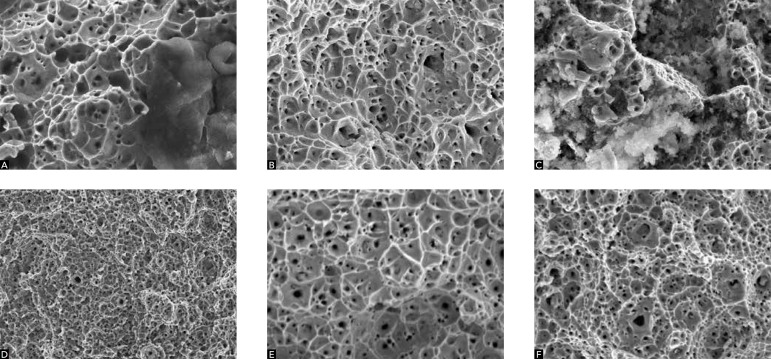
Surface morphology of fracture of superelastic NiTi and CuNiTi wires: A) NiTi superelastic by GAC; B) NiTi superelastic by TP; C) NiTi superelastic by Ormco; D) CuNiTi 27oC by Ormco; E) NiTi superelastic by Masel; F) NiTi superelastic by Morelli (X 2000).

**Figure 6:**
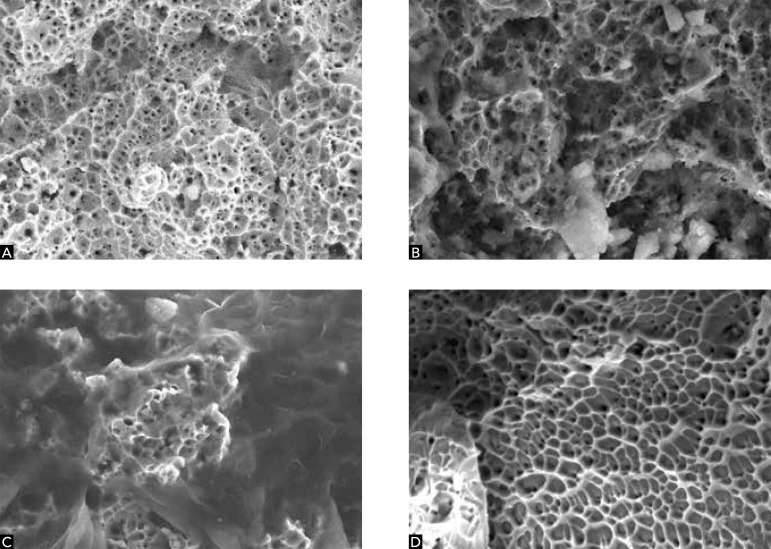
Surface morphology of fracture of heat-activated NiTi and CuNiTi wires: A) heat-activated NiTi by GAC; B) heat-activated NiTi by TP; C) CuNiTi 35oC by Ormco; D) heat-activated NiTi by Unitek (X 2000).

Figures 4 to 6 show the surface morphology of fractured NiTi and CuNiTi wires subjected to traction tests. The wires presented macroscopic plastic deformation with significant reduction in diameter. The fracture was cup-cone type, which is typical of high-ductility materials. This occurred to all subgroups of wires, implying that they underwent permanent deformations before fracturing, since they were subjected to tension and traction that were greater than their respective yield value. The analyses of fractured surfaces with greater increase (Figs 5 and 6) demonstrate the presence of microcavities, proving that the fracture is of ductile type.

## DISCUSSION

With regard to the chemical composition of the tested wires, we observed that, generally, the tested NiTi wires presented Ni and Ti as the main elements of the alloy with minor differences in their composition. We also observed the presence of other chemical elements, especially Al, Si and Ca. Superelastic wires (Masel) presented a slightly lower percentage of Ni and Ti associated with a significative percentage of aluminum oxide. Superelastic wires (Ormco) presented a slightly lower percentage of Ni with higher percentage of calcium. Fischer-Brandies et al^9^ suggested that all wires present inclusions, chemical immunogenicity and manufacturing residue, such as silicon oxide (SiO_2_) and aluminum oxide (Al_2_O_3_), which was also observed in the present work.

The present work demonstrated that CuNiTi wires have a significant percentage of copper (Cu) in addition to Ni and Ti in their composition, as reported by Fischer-Brandies et al.^9^ According to Parvizi and Rock,^13^ adding between 5 and 6% of copper in the composition of superelastic NiTi wires would increase mechanical resistance and reduce the percentage of permanent deformation after deactivation, all of which are considered favorable characteristics. However, such benefits would be associated with an increase in the transition temperature of these wires for values greater than the ones found intraorally. An addition of 0.5% of chromium would be made with the purpose of avoiding such an increase and, thus, maintain the temperature at around 27ºC. But, in the present work, no percentage of chromium was found in the composition of CuNiTi wires.

As for the surface morphology of wires, the manufacturing process is carried out by drawing. Drawing is the process in which wires with larger cross-sections (wire rod) pass through tools (spinnerets) to have their dimensions reduced, thus, being transformed into another wire with adequate diameter and which can be used for several purposes, for instance, as round, square and rectangle orthodontic wires. The presence and intensity of drawing marks on the surface of the wires depend on the period of use and on the spinneret finishing, on the alloy composition and on the thermodynamic state of the wire rod. Thus, the longer the period of use of the spinneret, the greater the chance of it making slots and imperfections on the surface of the wires during the process. The following is among the consequences of drawing marks: greater concentration of tension on the wires, reduction in the material capacity of being longitudinally deformed, reduction in the limit of traction resistance, increase in the coefficient of attrition and increase in biofilm adhesion.^7^

In the present work, superelastic wires presented smoother surfaces in relation to the heat-activated ones, as reported by Fischer-Brandies et al.^9^ Among the superelastic wires, those by Masel and Morelli (Figs 2E and 2F) presented the lowest wire-surface roughness, with evidence of polishment after the drawing process. TP and GAC wires (Figs 2A and 2B) presented intermediate wire-surface roughness. Superelastic NiTi and CuNiTi 27ºC wires by Ormco (Figs 2C and 2D) presented the greatest wire-surface roughness, with deep spinneret marks, which led us to believe that they had not been subjected to any chemical or mechanical treatment after drawing.

Among the heat-activated wires (Fig 3), those by GAC, TP and Unitek as well as the CuNiTi 35ºC by Ormco presented inadequate characteristics regarding wire-surface roughness, with very visible drawing marks and slots seen at any degree of magnification. Such marks were more evident and deeper in wires by Unitek and in the CuNiTi 35ºC by Ormco (Figs 3C and 3D). When those wires were evaluated under magnification of 2000x (Figs 3E and 3F), they presented, in addition to drawing marks and slots, microcavities formed due to pullout of particles, possibly of NiTi,^3^ which was also observed in superelastic NiTi and CuNiTi 27ºC wires by Ormco. NiTi^3^ precipitation depends on the amount of Ni in solid solution, since it could have been altered by the presence of copper in the composition of CuNiTi wires. Variations in 0.1% of Ni would already be significant to induce the formation and precipitation of NiTi4. As for the superelastic NiTi wires by Ormco, they presented a slightly lower percentage of Ni in comparison to the other tested NiTi wires (Table 2), which could have induced the formation of a higher percentage of precipitation and pullout of particles of NiTi4.

With regard to the morphology of the fracture region of superelastic and heat-activated wires seen under magnification of 2000x (Figs 5 and 6), we observed the presence of microcavities which are a characteristic of ductile fractures, with the largest microcavities being formed by pullout of particles, possibly of NiTi4. Additionally, we observed that, in general, the size of the microcavities found for superelastic NiTi wires was larger than that found for heat-activated NiTi wires. Among the superelastic NiTi wires, those that presented the smallest microcavities were the CuNiTi 27ºC ones (Fig 5D). Wires by GAC, TP, Masel and Morelli (Figs 5A, 5B, 5E and 5F) presented microcavities of similar sizes. Among the heat-activated wires, those by GAC and Unitek (Figs 6E and 6H) presented smaller microcavities in comparison to wires of other samples.

The results of the present work showed that CuNiTi 35ºC wires presented inadequate characteristics regarding superficial topography, which could predispose a higher coefficient of attrition during tooth movement. Damon,^6^ however, asserted that the sequential use of orthodontic wires of CuNiTi 35ºC 0.014", 0.014" X 0.025" and 0.018" X 0.025" in self-bonding brackets would significantly reduce the coefficient of attrition generated in conventional mechanics, which would be the key to a more efficient treatment, with significant reduction in the mean period of chair time, in the number of visits paid to the orthodontist and in patient's degree of discomfort.

Nevertheless, a new research should be carried out in order to evaluate the attrition of such wires in relation to other superelastic and heat-activated NiTi and CuNiTi wires, given that through scanning electron microscopy, the present work, in agreement to results obtained by Fischer-Brandies et al,^9^ proved that CuNiTi 35ºC wires by Ormco presented not only very visible drawing marks and slots seen under any degree of magnification, but also microcavities formed due to pullout of particles of NiTi4, which could generate a higher coefficient of attrition.

## CONCLUSION

1. With regard to chemical composition, the tested NiTi wires presented predominance of Ni and Ti with a small percentage of Al, Ca, and Si. CuNiTi wires presented a significant percentage of copper in addition to Ni and Ti in their compositions.
2. With regard to surface morphology, the superelastic NiTi wires by Masel and Morelli presented the lowest wire-surface roughness, while the superelastic and heat-activated NiTi and CuNiTi wires by Ormco presented the greatest wire-surface roughness.
3. With regard to the morphology of fracture regions, CuNiTi 27ºC wires by Ormco and the heat-activated wires by GAC and Unitek presented the smallest microcavities, while CuNiTi 35ºC by Ormco presented the largest.
4. All wires presented fractured surfaces under ductile traction.
5. CuNiTi 35ºC wires did not present better morphologic characteristics on surfaces and fracture regions in comparison to the other wires.
